# The Key Role of Thermal Relaxation Time on the Improved Generalized Bioheat Equation: Analytical Versus Simulated Numerical Approach

**DOI:** 10.3390/ma18153524

**Published:** 2025-07-27

**Authors:** Alexandra Maria Isabel Trefilov, Mihai Oane, Liviu Duta

**Affiliations:** National Institute for Laser, Plasma and Radiation Physics, 077125 Magurele, Romania; alexandra.trefilov@inflpr.ro (A.M.I.T.); mihai.oane@inflpr.ro (M.O.)

**Keywords:** bioheat equations, laser–skin tissue interaction, thermal relaxation time, analytical and numerical simulations, MATHEMATICA software, COMSOL simulation

## Abstract

The Pennes bioheat equation is the most widely used model for describing heat transfer in living tissue during thermal exposure. It is derived from the classical Fourier law of heat conduction and assumes energy exchange between blood vessels and surrounding tissues. The literature presents various numerical methods for solving the bioheat equation, with exact solutions developed for different boundary conditions and geometries. However, analytical models based on this framework are rarely reported. This study aims to develop an analytical three-dimensional model using MATHEMATICA software, with subsequent mathematical validation performed through COMSOL simulations, to characterize heat transfer in biological tissues induced by laser irradiation under various therapeutic conditions. The objective is to refine the conventional bioheat equation by introducing three key improvements: (a) incorporating a non-Fourier framework for the Pennes equation, thereby accounting for the relaxation time in thermal response; (b) integrating Dirac functions and the telegraph equation into the bioheat model to simulate localized point heating of diseased tissue; and (c) deriving a closed-form analytical solution for the Pennes equation in both its classical (Fourier-based) and improved (non-Fourier-based) formulations. This paper investigates the nuanced relationship between the relaxation time parameter in the telegraph equation and the thermal relaxation time employed in the bioheat transfer equation. Considering all these aspects, the optimal thermal relaxation time determined for these simulations was 1.16 s, while the investigated thermal exposure time ranged from 0.01 s to 120 s. This study introduces a generalized version of the model, providing a more realistic representation of heat exchange between biological tissue and blood flow by accounting for non-uniform temperature distribution. It is important to note that a reasonable agreement was observed between the two modeling approaches: analytical (MATHEMATICA) and numerical (COMSOL) simulations. As a result, this research paves the way for advancements in laser-based medical treatments and thermal therapies, ultimately contributing to more optimized therapeutic outcomes.

## 1. Introduction

Diagnosis, therapy, and surgery are well known medical procedures. However, the mechanics of these procedures in living tissue may not be fully understood. Before applying any medical procedure, it is better to understand the mechanical properties of materials and tissues by solving the constitutive equations. Therefore, numerical and analytical modeling and simulation play an important role in biomechanics. They either solve existing constitutive equations or assist in the determination of unknown constitutive equations [1].

Heat transfer and temperature variation within the human body represent fundamental and complex processes governed by the thermodynamic behavior of biological systems, their intrinsic thermal properties, and their response to external thermal stimuli [2].

Modeling heat-related phenomena, such as bioheat transfer in biological tissues, is of significant importance for the advancement of biomedical technologies, including thermotherapy. A recent focus in this field is laser-based thermotherapy, which offers a precise and effective alternative to conventional thermal treatment methods. This minimally invasive technique employs light at specific wavelengths to selectively ablate abnormal tissue, promote cellular regeneration, and alleviate pain and inflammation [3,4,5,6,7,8]. Lasers can generate highly intense, short-duration pulses of light that can be focused on a microscale spot, resulting in a high-power density. This characteristic makes them particularly suitable for selectively heating localized regions of tissue, enabling applications such as blood coagulation, tissue ablation, and highly precise surgical incisions in various clinical contexts [9,10].

Laser thermotherapy offers several advantages over conventional treatment methods, including shorter procedure durations, reduced collateral tissue damage, minimized bleeding, inflammation, and edema, and a lower risk of scarring. Additionally, it can alleviate post-operative pain, stimulate cellular activity, and promote tissue regeneration [11]. Given that laser therapy involves the application of laser radiation to the body’s surface, a thorough understanding of the temperature dynamics in living tissues during and after exposure is essential to ensure both the safety and therapeutic efficacy of the procedure.

Modeling laser thermotherapy phenomena is essential for treatment planning, the development and refinement of temperature monitoring technologies, and for gaining a comprehensive understanding of heat-related processes. These include bioheat transfer and its spatial distribution within living tissues, thermally induced mechanical responses, the extent of tissue damage, and heat-generated stress. Such models serve as critical tools for optimizing therapeutic protocols, minimizing adverse effects, and enhancing patient-specific treatment strategies.

Methods for predicting heat evolution in biological tissue during thermal treatments are essential for developing strategies to control thermal dose, optimize the performance parameters of heat-inducing medical devices, and minimize thermal damage to healthy tissue [12]. The first mathematical model describing heat transfer in soft human tissue during thermal exposure was introduced by Pennes in 1948, providing a framework for heat flow analysis in biological tissue, specifically in the forearm [13]. This study incorporated multiple phenomenological mechanisms, including metabolic heat generation, thermal conduction, radiation, blood perfusion, and phase changes.

To formulate the bioheat equation, Pennes made several key assumptions: (i) heat exchange between blood and surrounding tissue is primarily driven by arterial capillary blood flow; (ii) arterial blood entering the tissue capillaries at a relatively higher temperature (*T_b_*) is in thermal equilibrium with the adjacent tissue; and (iii) the volumetric blood perfusion rate, defined as the blood flow per unit volume of tissue, is considered constant.

Taking all the above hypotheses into consideration, the general form of the Pennes bioheat equation is expressed as

(1) $$\rho c\frac{\partial T}{\partial t}=k∆T+\rho_{b}c_{b}\omega_{b}\left(T_{b}-T\right)+E$$

where *ρ*, *c*, *T*, *t*, and *k* are the tissue density, specific heat capacity, thermal exposure time, and thermal conductivity, respectively. Parameters such as *ρ_b_*, *c_b_*, and *ω_b_* represent the blood density, specific heat, and volumetric perfusion rate, respectively, whilst *E* is defined as the thermal energy production.

$$E=Q_{r}+Q_{m}$$

where *Q_r_* represents the regional heat source and *Q_m_* denotes the metabolic heat production.

The mathematical model describing the thermal behavior of biological tissues, formulated by Pennes’ bioheat equation, has been widely used and adapted for specific applications, primarily through numerical methods and, to a lesser extent, analytical approaches. Numerous modifications of Pennes’ bioheat equation have been reported in the literature, each tailored to specific objectives, such as: modeling the thermomedical response of biological tissues to high-temperature exposure [14], analyzing heat transfer at tissue boundaries across different coordinate systems [15], investigating time-dependent multiregion thermal exposure [16], characterizing heat transfer behavior in biological tissues [17], and defining boundary conditions for medical treatments involving skin exposure to external heat sources [18]. The standard Pennes model, along with most studies in the literature, is based on the traditional Fourier assumption that heat propagation occurs instantaneously, implying an infinite propagation velocity. However, this assumption contradicts Einstein’s theory of relativity, particularly in systems with reduced dimensions. To address this limitation, Cattaneo [19] and Vernotte [20] modified the classical heat conduction model by incorporating the effect of thermal relaxation time, denoted as *τ_q_*. This parameter represents the finite time required for a thermally excited region to respond to a heat flux and is commonly defined as the time it takes for the temperature in a directly heated area to decrease to 37% of its initial rise [11].

The Cattaneo–Vernotte mathematical model introduces the concept that heat propagation occurs at a finite velocity, which decreases with increasing thermal relaxation time. This modification aligns with the observed phenomenon that heat propagates significantly slower in biological tissues than in most other materials, making thermal relaxation time a crucial parameter in bioheat transfer analysis [21]. Thus, the introduction of thermal relaxation time into the bioheat equation represents an essential first step toward incorporating principles of non-equilibrium irreversible thermodynamics. It accounts for the finite temporal lag between the imposition of a temperature gradient and the resulting heat flux. This formulation allows for finite-speed thermal wave propagation, yielding a more physically consistent description of spatiotemporal temperature evolution, particularly under conditions involving short exposure durations, highly localized energy deposition or rapid heating scenarios [22,23]. Furthermore, since the Cattaneo–Vernotte formulation accounts for a finite thermal propagation speed, it reflects more realistic heat transfer dynamics in biological and solid materials.

Zhukovsky considers the Cattaneo, Fourier, and Guyer–Krumhansl heat conduction laws and proposes a comprehensive heat transport model that incorporates the telegraph equation to analyze both heat conduction and thermal wave propagation phenomena [24]. One of the most significant characteristics of the solutions to the Zhukovsky heat equations is the strong dependence on the temporal variable; specifically, the shorter the time interval considered, the more rigorous and accurate the solutions become. Mathematically, the telegraph equation represents a second-order hyperbolic partial differential equation, in contrast to the parabolic nature of the classical Fourier conduction model. In this regard, the telegraph-based non-Fourier model provides a more realistic theoretical and computational framework for modeling thermal energy transport in biological tissues—especially under laser-therapeutic conditions involving high spatial resolution and brief heating intervals.

This is commonly referred to as high-temperature thermal therapy. The small, localized region of diseased tissue targeted by the laser beam, known as point heating, is mathematically represented using the Dirac function. This approach ensures that healthy tissue remains unaffected by the photo-thermal effect. Furthermore, by incorporating the telegraph equation, the bioheat equation is further refined, allowing for the inclusion of additional parameters that can be easily modified or adjusted, thereby enhancing the model’s flexibility and accuracy.

A three-dimensional (3D) analytical model was developed using the improved bioheat equation mentioned above, incorporating the analysis of thermal relaxation time to describe laser–diseased tissue interactions. Two formulations of the improved bioheat equation were derived and modeled: (i) the classical Pennes equation, based on Fourier’s law, and (ii) the generalized non-Fourier bioheat equation. This approach enables a more generalized and realistic representation of heat exchange between biological tissue and blood flow by predicting non-uniform temperature distributions.

The proposed simulations also account for a critical yet often overlooked parameter in laser therapy: the laser wavelength. This parameter significantly influences the interaction between laser irradiation and biological tissues, as absorption characteristics vary with wavelength, enabling a wide range of medical applications. For example, wavelengths with high absorption in water are particularly effective for cutting or vaporizing tissue. Consequently, the laser wavelength directly affects the penetration depth of the radiation, thereby determining the spatial distribution of heat, the direction of thermal propagation, and ultimately the thickness of the necrotic zone [25].

The simulation considers a near-infrared laser irradiation wavelength range of 700–900 nm, corresponding to the optical therapeutic window, which enables maximal laser light penetration into biological tissues. This spectral window is particularly advantageous for biomedical applications due to reduced absorption by water and hemoglobin, facilitating efficient subsurface heating while minimizing surface damage. Within this wavelength range (700–900 nm), photons are predominantly absorbed or undergo multiple scattering events that rapidly randomize their propagation directions. As a result, the influence of tissue-specific optical heterogeneities is significantly reduced. This attenuation of directional bias diminishes the effects of initial structural anisotropy, thereby facilitating deeper energy penetration [25,26,27,28,29,30]. Although some degree of optical anisotropy may arise from the alignment of collagen fibers, its impact on macroscopic heat distribution remains minimal. Under the experimental conditions considered in this study, optically induced thermal transport is governed primarily by the tissue’s absorption characteristics and intrinsic thermal properties.

To achieve mathematical validation of the simulation results, the study is structured into two main parts: (a) the first part investigates the effect of laser-induced heat generation on the diseased skin tissue and adjacent subcutaneous tissue surface using both the classical and modified Pennes bioheat equations, implemented in MATHEMATHICA software; and (b) the second part evaluates the resulting thermal distribution by applying the same bioheat equation parameters within the COMSOL Multiphysics environment for comparison. Accordingly, the current study provides a robust theoretical foundation that can inform and guide future experimental investigations.

MATHEMATICA is a computational platform capable of performing analytical simulations across all modes of heat transfer, making it particularly suitable for characterizing laser-induced heat propagation in biological tissues under various therapeutic conditions. Its advanced symbolic computation capabilities allow for the development of innovative solution techniques, enabling more realistic representations of heat exchange between biological tissue and blood flow by capturing non-uniform temperature distributions. Furthermore, each simulation was completed in under one minute on a standard commercial PC equipped with an Intel i7 processor [31], demonstrating both the efficiency and practicality of the approach.

In comparison, COMSOL Multiphysics provides a dedicated heat transfer module that enables a more detailed, complex, and robust numerical analysis of both the classical and improved bioheat equations. Utilizing the Finite Difference Method, the software simulates thermal distribution by assigning specific thermal conductivity properties to each tissue layer, thereby capturing spatial variations in heat propagation with higher accuracy. Simulations were conducted using the Heat Transfer Module in COMSOL Multiphysics to investigate the influence of heating and cooling processes, as well as the evolution of bioheat transfer in tissues irradiated by external radiation sources such as lasers. This module facilitates the development of a comprehensive Multiphysics model that accounts for the coupling between various physical phenomena, including: (i) absorption, emission, and scattering of radiation by blood and tissue; (ii) heat transfer within biological tissues; (iii) thermal conduction and heat storage in living tissues; (iv) thermal damage and associated temperature thresholds; and (v) temperature-dependent damage fraction. Despite its versatility and accuracy, the software requires significant computational resources. It is highly demanding on RAM and CPU, often necessitating the use of high-performance computers. Additionally, simulation runtimes can be considerably longer compared to analytical approaches.

Such simulations are particularly significant for tumor treatment, where irregular blood vessel growth and structurally heterogeneous tissue organization complicate thermal response prediction. Modeling was chosen for both practical and ethical considerations, as it allows for detailed analysis while minimizing the risks associated with direct human exposure to laser radiation. Consequently, this research opens new avenues for laser-based medical treatments, potentially leading to more optimized and effective therapeutic outcomes.

## 2. Constitutive Equations and Mathematical Formalism

It is important to recognize that a wide range of Pennes-type bioheat equations currently exists in the literature. In the present study, we selected Ref. [1] as the starting point for our model, specifically adopting the form presented in Equation (1) of the indicated reference. Moreover, Equation (12) from the same source [1] inspired our formulation of the heat source term *Q* as a product of Dirac delta functions, allowing us to model localized energy deposition analytically.

In developing our non-Fourier extension of the Pennes bioheat model, we explicitly followed the thermodynamic principles of Extended Irreversible Thermodynamics (EIT), as formulated by Jou et al. [32]. Specifically: (i) the introduction of a finite thermal relaxation time *τ_q_* ensures compliance with causality by enabling a finite propagation speed of thermal waves, (ii) the model maintains positive entropy production, thereby satisfying the second law of thermodynamics, and (iii) the resulting telegraph-type (hyperbolic) formulation is mathematically equivalent to the Cattaneo–Vernotte equation, which is derived within the EIT framework. It is important to emphasize once again that the core feature of the Cattaneo–Vernotte solutions is the finite speed of thermal propagation. This behavior is consistent with physical “reality” as described by Einstein’s special theory of relativity, which imposes a finite speed limit for the transmission of physical signals. In this regard, several assumptions are made to define the physical system for the modified bioheat equation: (i) blood perfusion in the skin remains constant throughout the heating process; (ii) metabolic heat contributions to the total energy balance are considered negligible; and (iii) the tissue is treated as a homogeneous and infinitely large heat-conducting medium.

Another assumption presented in the study relates to adopting an isotropic optical approximation. This is despite the fact that the skin exhibits pronounced mechanical anisotropy, characterized by direction-dependent elasticity and tensile strength. This is primarily due to the preferential alignment of collagen fibers within the dermis [33,34]. However, the present study is focused on the simulation of optically induced thermal transport in skin tissue, modeled through the Pennes bioheat equation. In this context, the dominant parameters influencing heat transfer are the optical absorption coefficient, the reduced scattering coefficient, and the thermal properties of the tissue (e.g., thermal conductivity and specific heat capacity). Our simulations—performed using both MATHEMATICA and COMSOL Multiphysics—are designed to predict the spatiotemporal evolution of tissue temperature in response to near-infrared laser irradiation (700–900 nm).

At the considered wavelengths, although a certain degree of optical anisotropy may result from the alignment of collagen fibers, its influence on macroscopic heat distribution remains minimal. Therefore, adopting an isotropic optical approximation is considered justified for the following reasons:

(i) Multiple scattering mitigates optical anisotropy: In the near-infrared range, photons undergo numerous scattering events, which rapidly randomize their propagation directions. This process significantly reduces the influence of initial structural anisotropy on the overall photon fluence distribution [29,30];
(ii) Use of bulk optical coefficients: The optical properties employed in our model (e.g., absorption and reduced scattering coefficients, µ) are widely accepted for thermal modeling of skin and other biological tissues in the NIR range. These parameters inherently account for tissue heterogeneities and average fiber orientations and are commonly utilized in deterministic simulations [29];
(iii) Dominance of thermal diffusion: Once heat is generated via optical absorption, the subsequent temperature distribution is primarily governed by thermal conduction. At the macroscopic level, thermal conductivity in skin is generally treated as isotropic, with any anisotropy being relatively minor compared to mechanical anisotropy [35].

Last, but not least, one should emphasize that Zhukovsky has developed a comprehensive state-of-the-art theoretical framework of differential equations (DE) in non-homogeneous cases [24]. The core idea underlying Zhukovsky’s treatment of non-homogeneous DE is that the non-homogeneous term can be formally separated from the homogeneous part of the equation. In this context, the non-homogeneous component can be interpreted as a temporal source term, typically considered at the initial time point (*t* = 0). This separation allows the homogeneous and non-homogeneous dynamics to be analyzed with greater clarity and mathematical tractability. These assumptions and their implications were clearly demonstrated in Ref. [36], where the methodology was applied and validated.

Under these assumptions, the bioheat equation simplifies to

(2) $$\frac{\partial^{2}T}{\partial t^{2}}+\left(\rho c+\rho_{b}c_{b}\omega_{b}\tau_{q}\right)\frac{\partial T}{\partial t}+\rho_{b}c_{b}\omega_{b}T=k\left(\frac{\partial^{2}T}{{\partial x}^{2}}+\frac{\partial^{2}T}{{\partial y}^{2}}+\frac{\partial^{2}T}{{\partial z}^{2}}\right)+Q_{met}+Q_{ext}\left(x,y,z,t\right)+\rho_{b}c_{b}\omega_{b}T_{b}$$

(3) $$\left(\frac{1}{\rho c+\rho_{b}c_{b}\omega_{b}\tau_{q}}\right)\frac{\partial^{2}T}{\partial t^{2}}+\frac{\partial T}{\partial t}+\left(\frac{\rho_{b}c_{b}\omega_{b}}{\rho c+\rho_{b}c_{b}\omega_{b}\tau_{q}}\right)T=\left(\frac{k}{\rho c+\rho_{b}c_{b}\omega_{b}\tau_{q}}\right)\left(\frac{\partial^{2}T}{{\partial x}^{2}}+\frac{\partial^{2}T}{{\partial y}^{2}}+\frac{\partial^{2}T}{{\partial z}^{2}}\right)\;{+\;(Q}_{met}+\;Q_{ext}\;\left(x,y,z,t\right)+\rho_{b}c_{b}\omega_{b}T_{b})/\;\left(\rho c\;{+\rho }_{b}c_{b}\omega_{b}\tau_{q}\right)$$

and therefore

(4) $$\left(\frac{1}{\rho c+\rho_{b}c_{b}\omega_{b}\tau_{q}}\right)\frac{\partial^{2}T}{\partial t^{2}}+\frac{\partial T}{\partial t}+\left(\frac{\rho_{b}c_{b}\omega_{b}}{\rho c+\rho_{b}c_{b}\omega_{b}\tau_{q}}\right)T=\left(\frac{k}{\rho c+\rho_{b}c_{b}\omega_{b}\tau_{q}}\right)\left(\frac{\partial^{2}T}{{\partial x}^{2}}+\frac{\partial^{2}T}{{\partial y}^{2}}+\frac{\partial^{2}T}{{\partial z}^{2}}\right)+Q\;\left(x,y,z,t\right)/\;\left(\rho c\;{+\rho }_{b}c_{b}\omega_{b}\tau_{q}\right)$$

because

(5) $$Q_{met}+\rho_{b}c_{b}\omega_{b}T_{b}\ll Q_{ext}\left(x,y,z,t\right)=Q\left(x,y,z,t\right)$$

where *ρ* is the tissue density; *c* represents the specific heat capacity of the tissue; *T* denotes the tissue temperature; *t* is the thermal exposure time, *ρ_b_* is the blood density; *c_b_* represents the specific heat capacity of blood; *τ_q_* is the thermal relaxation time; *ω_b_* stands for the blood perfusion rate; *T_b_* is the blood temperature; *k* is the thermal conductivity of the tissue; *x* represents the heat transmission distance; *Q_met_* is the metabolic heat generation rate per unit volume of the tissue, and *Q_ext_* is the distributed volumetric heat source due to laser heating of the tissue.

The *τ_q_* parameter describes the tissue’s response to thermal perturbation. In most materials, *τ_q_* typically ranges from 10^−14^ to 10^−8^ s. However, in biological tissues, it is significantly longer, ranging from a few seconds to several tens of seconds [37]. In Equation (1), when *τ* approaches 0, the equation simplifies to the traditional Pennes bioheat equation.

The temperature rise in the tissue induced by laser devices used in clinical applications is governed by the equation describing the heat generation in tissue, denoted as *Q* [1]:

(6) $$Q\left(x,y,z,t\right)\;=\;P\left(t\right)\delta \left(x\right)\delta (y)\delta (z)e^{{-\alpha }_{abs}x}≅P_{0}\delta (x)\delta (y)\delta (z)$$

where *P(t)* denotes the strength of the point heating source, which may vary over time; δ represents the Dirac function; (0, 0, 0) specifies the location of the point heating source, and α_abs_ is the absorption coefficient of laser radiation in the tissue.

One should emphasize that the Dirac delta function models the spatial localization and immediacy of energy input, while the telegraph equation introduces a finite temporal response of heat conduction. Together, these features form a cohesive framework for modeling thermal processes in laser-tissue interactions with enhanced physical realism. Another important aspect is that when the heat source is represented by a Dirac delta function, the Zhukovsky equations admit closed-form analytical solutions.

In this study, we investigate the thermal response of biological tissue to three-point heating and analyze the consequent effects of temperature variations on the resulting stress and displacement within the tissue. For simplicity, we assume 0 < x << 1. Due to this assumption and to the ultrashort duration of the laser pulses, the propagation characteristics of thermal waves are expected to deviate from classical diffusion behavior. Under these specific conditions, thermal transport is dominated by localized and rapid energy deposition, while the spatial propagation of thermal disturbances becomes less pronounced.

Among the three-point heating sources, the heating strength is considered identical with the laser power density (irradiance): *P(t)* = 1.0 × 10^7^ W m^−3^ [1].

The telegraph equation, also known as the hyperbolic heat equation, is considered due to its enhanced parameter flexibility. The hyperbolic bioheat equation is formulated using a telegraph-type model, which introduces a finite thermal relaxation time (τ_q_) to account for the delayed response of heat flux to thermal gradients. This formulation enables the description of thermal wave propagation with finite speed, providing a more physically accurate representation of transient and localized thermal phenomena. It is expressed in the following form:

(7) $${(\tau \partial }_{t}^{2}+\partial_{t})T\left(x,t\right)\;=\left(D\cdot \nabla^{2}+\mu \right)T(x,t)$$

where *D* denotes the thermal diffusivity of the tissue, *μ* represents a linear attenuation coefficient, and τ is the standard relaxation time in the telegraph equation, which is related to the thermal relaxation time through Equation (8).

A comparison between Equations (3) and (7) allows for the identification of the following relationships:

(8) $$\tau \;\equiv \;\left(\frac{\rho c\tau_{q}}{\rho c\;+\;\rho_{b}c_{b}\omega_{b}\tau_{q}}\right)$$

(9) $$D\;\equiv \;\left(\frac{k}{\rho c+\rho_{b}c_{b}\omega_{b}\tau_{q}}\right)$$

(10) $$\mu \;\equiv -\frac{\rho_{b}c_{b}\omega_{q}}{\rho c+\rho_{b}c_{b}\omega_{b}\tau_{q}}$$

From the mathematical analogy between the heat and telegraph equations [24], it can be deduced that

(11) $$T\left(x,y,z,0\right)\propto \frac{Q\left(x,y,z,t\right)}{\left(\rho c{+\rho }_{b}c_{b}\omega_{b}\tau_{q}\right)\;\;}=\;\frac{P\delta \left(x\right)\delta \left(y\right)\delta \left(z\right)}{\left(\rho c\;{+\rho }_{b}c_{b}\omega_{b}\tau_{q}\right)\;\;}$$

Thus, the resulting telegraph heat equation has the following solution [24]:

(12) Tx,y,z,t = e−εt2·t32π2α32·ωv·K2(2ωv)

where *ω_b_* represents the tissue perfusion rate, and *K_2_* denotes the modified Bessel function of the second kind.

(13) $$\omega =\varepsilon^{2}+4µ\mathrm{and}\;v=\frac{t^{2}-(x^{2}+y^{2}+z^{2})/\alpha }{16}$$

We identify the following relationships:

(14) ε = 1/τ;     α = D/τ;       k = μ/τ

The final solution of the bioheat equation can be expressed as follows:

(15) $$\begin{array}{c} T\left(x,y,z,t\right)=e^{-\frac{-\left(\rho c\;+\rho_{b}c_{b}\omega_{b}\tau_{q}\right)\cdot t}{2\rho c\tau_{q}}}\cdot \frac{t}{32\pi^{2}\cdot {\left(\left(\frac{k}{\rho c+\rho_{b}c_{b}\omega_{b}\tau_{q}}\right)/\left(\frac{\rho c\tau_{q}}{\rho c+\rho_{b}c_{b}\omega_{b}\tau_{q}}\right)\right)}^{1.5}}\cdot \\ \left(\frac{\left({\left(\frac{\rho c\tau_{q}}{\rho c+\rho_{b}c_{b}\omega_{b}\tau_{q}}\right)}^{2}-4\cdot \frac{\rho_{b}c_{b}\omega_{q}}{\rho c+\rho_{b}c_{b}\omega_{b}\tau_{q}}\right)}{\frac{t^{2}-(x^{2}+y^{2}+z^{2})/\left(\left(\frac{k}{\rho c+\rho_{b}c_{b}\omega_{b}\tau_{q}}\right)/\left(\frac{\rho c\tau_{q}}{\rho c+\rho_{b}c_{b}\omega_{b}\tau_{q}}\right)\right)}{16}}\right)\cdot K_{2}(4({\left(\frac{\rho c\tau_{q}}{\rho c+\rho_{b}c_{b}\omega_{b}\tau_{q}}\right)}^{2}-4\cdot \\ \frac{\rho_{b}c_{b}\omega_{q}}{\rho c+\rho_{b}c_{b}\omega_{b}\tau_{q}}){\left(\frac{t^{2}-(x^{2}+y^{2}+z^{2})/\left(\left(\frac{k}{\rho c+\rho_{b}c_{b}\omega_{b}\tau_{q}}\right)/\left(\frac{\rho c\tau_{q}}{\rho c+\rho_{b}c_{b}\omega_{b}\tau_{q}}\right)\right)}{16}\right))}^{0.5} \end{array}$$

For a comprehensive understanding and accurate calculation of thermal relaxation time, one may refer to the following reference [38], which states

(16) $$\tau_{q}=\frac{d^{2}}{16D}$$

where *d* is the target diameter (in millimeters) and *D* is the tissue diffusivity (in square millimeters per second).

Table 1 provides an overview of the main physical parameters employed in all computational analysis throughout this study.

## 3. Results and Discussion of Simulation Models

Thermal relaxation time represents a critical parameter influencing heat dissipation within biological tissues during laser exposure. Thus, simulating the laser–skin interaction as a function of thermal relaxation time allows for a precise assessment of thermal damage thresholds, the optimization of laser parameters, and improved understanding of tissue response dynamics. By incorporating thermal relaxation time into the simulations, one can predict tissue heating and cooling more accurately, thereby enhancing the safety, efficacy, and clinical relevance of laser-based dermatological procedures.

**Figure 1:**
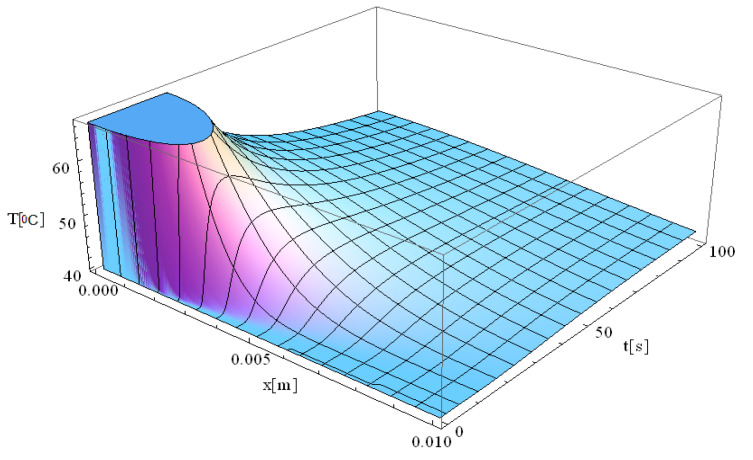
Simulated temperature distribution in laser-irradiated diseased tissue as a function of the spatial coordinate along the laser propagation direction (*x*) and the thermal exposure time (*t*). The computational domain assumes a target diameter (*d*) of 2 mm, with fixed spatial coordinates *y* = *z* = 0, and a thermal relaxation time $\left(\tau_{q}\right)\;of\;0$ s, corresponding to a classical Fourier heat conduction regime.

**Figure 2:**
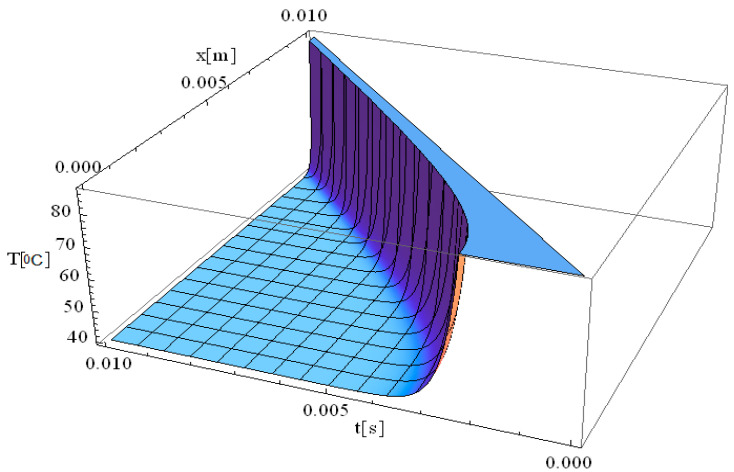
Simulated temperature distribution in laser-irradiated diseased tissue as a function of the spatial coordinate along the laser propagation direction (*x*) and the thermal exposure time (*t*). The computational domain assumes a target diameter (*d*) of 2 mm, with *z* = 0, and a thermal relaxation time $\left(\tau_{q}\right)$ of 1.16 s.

**Figure 3:**
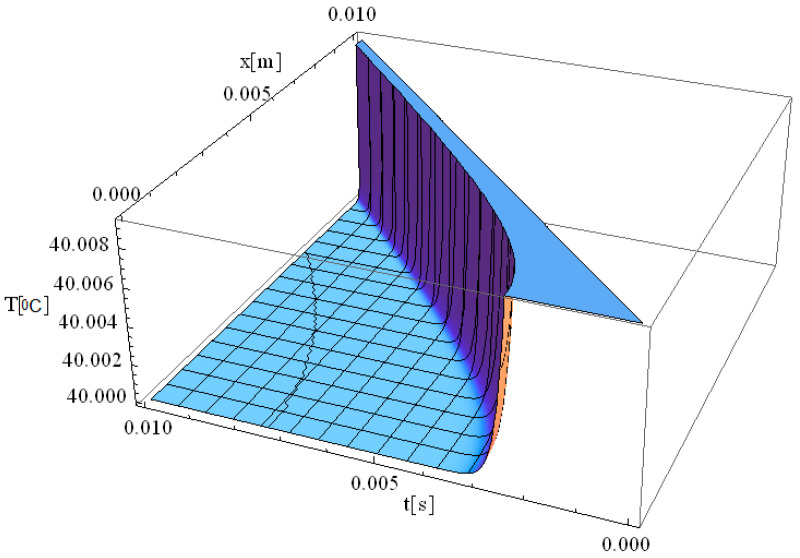
Simulated temperature distribution in laser-irradiated diseased tissue as a function of the spatial coordinate along the laser propagation direction (*x*) and the thermal exposure time (*t*). The computational domain assumes a target diameter (*d*) of 3 mm, with *z* = 0, and a thermal relaxation time $\left(\tau_{q}\right)$ of 2.61 s.

**Figure 4:**
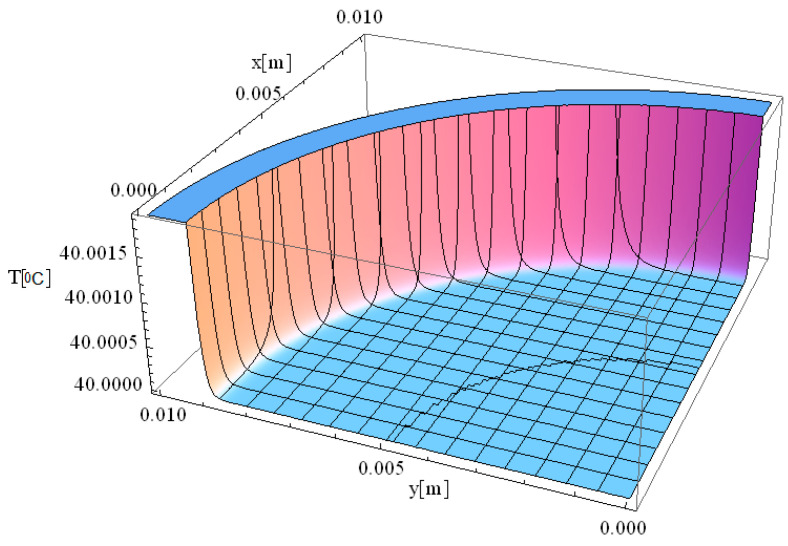
Simulated temperature distribution in laser-irradiated diseased tissue as a function of the spatial coordinates (*x*,*y*). The computational domain assumes a target diameter (*d*) of 3 mm, with *z* = 0, *t* = 0.01 s, and a thermal relaxation time $\left(\tau_{q}\right)$ of 2.61 s.

**Figure 5:**
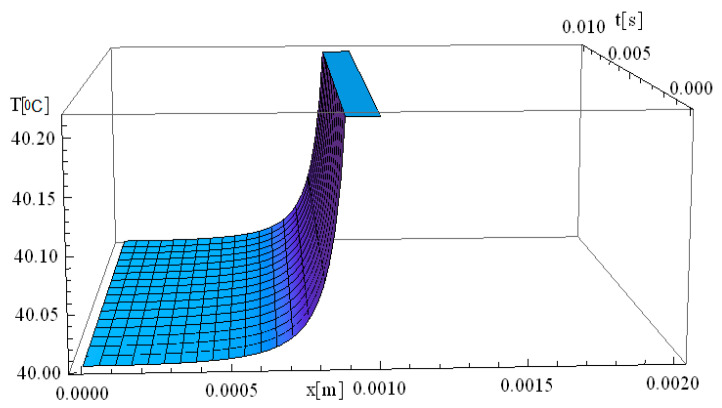
Simulated temperature distribution in laser-irradiated diseased tissue as a function of the spatial coordinate along the laser propagation direction (*x*) and the thermal exposure time (*t*). The computational domain assumes a target diameter (*d*) of 3 mm and a thermal relaxation time ${(\tau }_{q})$ of 0.01 s.

**Figure 6:**
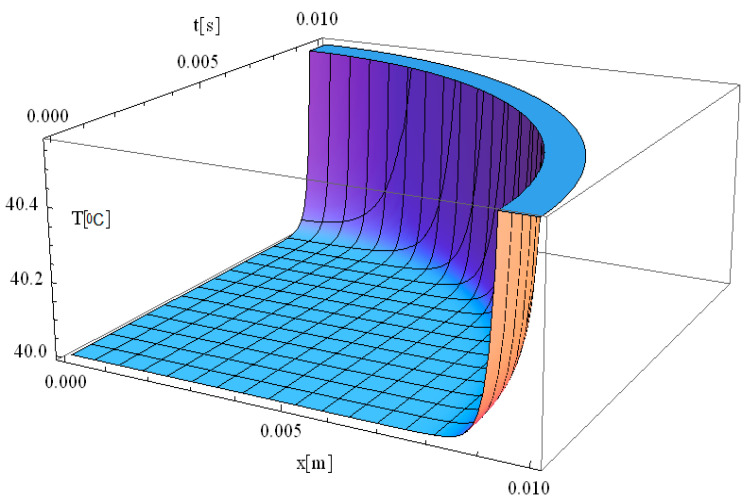
Simulated temperature distribution in laser-irradiated diseased tissue as a function of the spatial coordinate along the laser propagation direction (*x*) and the thermal exposure time (*t*). The computational domain assumes a target diameter (*d*) of 2 mm, with *z* = 0.001, and the thermal relaxation time ($\tau_{q})$ of 1.16 s.

In this respect, Figure 1, Figure 2, Figure 3, Figure 4, Figure 5 and Figure 6 illustrate 3D models depicting the thermal field (temperature distribution) resulting from laser-skin tissue interaction as a function of thermal relaxation time. The interaction between laser electromagnetic radiation and biological tissue was simulated using MATHEMATICA software (version 11.0).

The computational domain is defined within a rectangular coordinate system, with the heat source positioned at the origin. The spatial coordinate system (*x*, *y*, *z*) represents arbitrary positions on the diseased tissue surface. The *x*-axis denotes the spatial direction in which the laser propagates, while the *y*- and *z*-axes are oriented perpendicular to it. This setup allows for an accurate representation of heat diffusion and thermal wave propagation in the biological tissue.

The boundary conditions corresponding to Equation (12), as applied within the computational domain, are illustrated in Figure 1, Figure 2, Figure 3, Figure 4, Figure 5 and Figure 6. These boundary conditions define the thermal constraints imposed on the model, thereby ensuring an accurate simulation of heat transfer phenomena and laser–tissue interactions.

In our analytical model (MATHEMATICA), the laser heating source was represented using a Dirac delta function, which serves as an idealized point source located at the laser focus. This mathematical abstraction enables closed-form solutions and provides valuable insight into the fundamental mechanisms of thermal propagation under highly localized energy deposition.

To facilitate Guyer–Krumhansl heat conduction and gain a clearer understanding of the differences among the following simulations, a comparative table highlighting the main distinctions has been included (Table 2).

It is important to note that the inclusion of τ_q_ values such as 1.16 s or 2.61 s in our simulations is not only mathematically coherent but also thermodynamically well-founded, in accordance with the theoretical basis established by Jou et al. [32].

Figure 1 illustrates the simulation of thermal field evolution as a function of both space (*x*—spatial direction of laser propagation) and time (*t*—thermal exposure time, which implies the total time of tissue exposure at a given temperature). The simulation considers a target diameter (*d*) of 2 mm, with the spatial coordinates set at *y* = *z* = 0 m, and assumes a thermal relaxation time ($\tau_{q})$ of 0 s. This simulation is based on the derived analytical formula of the classical Pennes equation. It should be noted that the formalism used to solve the Pennes differential equation elegantly accommodates solutions in which the temperature *T* is non-zero for any given value of *x* and *t*.

At the moment the laser beam interacts with the tissue (very close to [x,t] = [0]), an immediate temperature rise occurs at the laser focal point. Due to the assumption of infinite heat propagation velocity in the classical Pennes model, heat diffusion occurs instantaneously throughout the domain. This results in an abrupt temperature gradient at the point of laser incidence, where the maximum temperature is reached. One should also note that the high temperatures diffuse into the immediately surrounding tissue layers up to 7 mm.

The temperature remains elevated for approximately 30 s, after which a gradual relaxation process occurs until the baseline temperature of 40 °C is restored. This observation suggests that the tissue directly exposed to laser heating is more susceptible to thermal damage compared to adjacent regions that are not directly irradiated. The localized heating effect aligns with findings in the literature on laser therapy [41,45], where selective thermal damage is achieved, ensuring that diseased tissue is effectively treated while minimizing harm to surrounding healthy tissue. This controlled thermal response is a key advantage of laser-based medical treatments (i.e., hadron-therapy), contributing to precise and efficient therapeutic outcomes.

Figure 2 illustrates the simulation of temperature distribution as a function of both the spatial coordinate along the laser propagation direction (*x*) and the thermal exposure time (*t*), incorporating the effects of thermal relaxation time. Compared to the classical Pennes equation, which assumes instantaneous heat diffusion, the inclusion of thermal relaxation effects modifies the temperature evolution, resulting in a more physically accurate thermal response. The simulation in Figure 2 exhibits the same thermal behavior as that observed in Figure 1 at the origin, very close from a mathematical perspective at the point [x,t] = [0]. A key observation in Figure 2 is the immediate rise in temperature at the laser interaction site, where temperatures reach values as high as 90 °C. This behavior reflects the localized absorption of laser energy at the focal point, leading to a rapid thermal response. Regarding the thermal field behavior along the *x*-axis, it is observed that the temperature gradient decreases as distance increases, reaching up to 10 mm. This suggests that thermal conduction is not uniform, and heat transport is influenced by both conduction and relaxation effects. The thermal relaxation time plays a crucial role in modulating how quickly heat spreads, preventing unrealistic instantaneous temperature increases in distant regions.

The thermal propagation distance is directly dependent on the exposure time, meaning that by increasing exposure duration to 0.01 s, the thermal field propagation distance can extend further into the tissue, up to 10 mm. However, this thermal propagation remains tightly controlled by controlling the exposure time, thereby allowing, for example, clinicians to precisely limit the damage area and reduce the chance of affecting surrounding healthy tissue. This property is particularly advantageous in laser therapy applications, where targeted destruction of diseased tissue is required while preserving adjacent structures.

Furthermore, considering the relaxation time of the laser-tissue interaction, it is evident that non-Fourier heat conduction models provide a more accurate computation of temperature distribution, particularly when deep laser penetration is needed to reach and destroy diseased areas. This highlights the importance of incorporating thermal relaxation effects into bioheat transfer models to enhance the precision of laser-based medical treatments.

Figure 3 illustrates the simulation of the thermal field as a function of the spatial coordinate along the laser propagation direction (*x*) and the thermal exposure time (*t*). The computational domain considers a target diameter (*d*) of 3 mm, and $\tau_{q}$ of 2.61 s, with *z* = 0. Compared to previous cases, this simulation provides insights into how increasing the laser spot size influences thermal field dynamics and the relaxation process in biological tissue.

When the target diameter is increased to 3 mm, the thermal relaxation time approximately doubles, leading to a slower heat propagation process. Consequently, the thermal field intensity decreases, with the maximum temperature at the origin reaching up to 41 °C, which is significantly lower than the 90 °C observed in Figure 2. This indicates that when the laser spot diameter is 3 mm, the energy is distributed over a larger area, resulting in a more uniform heating profile with less localized thermal accumulation. The extended relaxation time (*τ_q_* = 2.61 s) further modulates the rate of temperature increase and heat propagation, allowing for a gradual and controlled temperature rise rather than an abrupt spike. In summary, by adjusting the diameter of the laser spot, the temperature transfer and distribution can be precisely controlled, allowing for an optimized thermal response in biological tissue. These findings provide reliable guidelines for controlling thermal tissue damage (i.e., reducing the likelihood of overheating localized tissue regions) while maintaining effective thermal treatment for specific clinical laser-based therapies.

Figure 4 illustrates the simulation of thermal field distribution in the spatial coordinates [*x*, *y*], with thermal maxima observed at [0, 0.01] and at [0.01, 0]. This simulation provides valuable insight into heat propagation behavior in biological tissue when exposed to localized laser heating. The observed temperature distribution follows a well-defined spatial gradient, which is essential for understanding heat diffusion dynamics and optimizing laser-based medical treatments.

The minimal variation of the thermal field observed in Figure 4 indicates that temperature peaks remain highly localized, confirming that heat distribution is effectively controlled within a confined area. The controlled thermal propagation in this simulation is ideal for the selective destruction of targeted tissues while minimizing unintended damage to adjacent regions. The smooth thermal gradient observed along both the *x*- and *y*-axes suggests that heat dissipation occurs gradually rather than abruptly, which is beneficial for avoiding excessive thermal stress in surrounding healthy tissue. The significance of this result lies in its potential applications in precise tissue ablation, such as in laser surgery and tumor treatment. The ability to accurately model temperature distribution allows for the optimization of laser parameters, including wavelength, intensity, and exposure time, ensuring effective tissue removal with minimal collateral damage. Furthermore, understanding spatial thermal behavior is critical for applications like dermatological treatments, selective photothermal therapy, and laser-assisted wound healing, where temperature regulation plays a key role in treatment efficacy and patient safety.

We would like to emphasize that Figure 2, Figure 3 and Figure 4 are fully consistent with the theoretical framework proposed by Zhukovsky [24] and illustrate the expected behavior, as derived from his model.

Figure 5 illustrates the simulation of temperature distribution as a function of penetration depth (*x*) and time (*t*), considering a short thermal relaxation time of 0.01 s. The results indicate that the thermal field remains stable over time, with the maximum temperature observed at a penetration depth of approximately 1 mm.

Since thermal propagation distance is minimal and invariant in space, this configuration is particularly well-suited for laser-assisted skin cancer treatments. The short thermal relaxation time (*τ_q_* = 0.01 s) contributes to rapid and efficient laser energy absorption, allowing for immediate therapeutic effects without excessive thermal spread. Moreover, the localized heating prevents deep tissue penetration, minimizing unintended necrosis and accelerating post-treatment healing.

Figure 6 illustrates the simulation of the thermal field in a 3D spatial format. This simulation shares the same thermal parameters as Figure 2, with the key difference being that *z* ≠ 0, allowing for an analysis of heat propagation in the *z*-direction, in addition to the previously studied *x*-direction.

When assigning values to the *z*-axis, the simulation allows for a more comprehensive examination of heat distribution within the tissue volume rather than just along a simple plane. The thermal field exhibits a smooth propagation pattern, with the highest temperature localized at the laser interaction site. As heat diffuses outward, a gradual temperature gradient forms, decreasing in both the *z*- and *y*-directions as energy dissipates. This behavior is particularly significant when considering non-uniform tissue structures, where heterogeneities in thermal conductivity, density, and blood perfusion influence heat transfer. The inclusion of the *z*-component in the simulation provides a more realistic representation of thermal diffusion in biological tissues, which is critical for accurate modeling of laser-assisted medical treatments. Thus, the 3D spatial simulation in Figure 6 is particularly valuable for optimizing laser-based therapeutic techniques, such as tumor ablation, dermatological treatments, and laser surgery. By considering thermal variations along the *z*-axis, clinicians can better estimate depth-dependent temperature profiles, which are crucial for selecting appropriate laser parameters (e.g., wavelength, intensity, and pulse duration) to ensure targeted tissue destruction while minimizing collateral effects.

These simulation results are consistent with the experimental findings reported by Museux et al. [46] concerning thermal exposure time and temperature evolution. In their in vivo experiments involving laser-induced damage (808 nm wavelength and similar irradiance), they observed that longer exposure times result in higher temperatures, which decrease nonlinearly as exposure duration is reduced.

In agreement with their findings (e.g., *t* = 20 s and T = 65.5 °C), the simulation presented in Figure 1 indicates a maximum temperature of approximately 70 °C at an extended exposure time of 30 s. As in their study, this temperature rise is followed by a gradual thermal relaxation process lasting about 50 s, during which the temperature returns to the baseline value of 40 °C.

However, when the exposure time is reduced to approximately 0.01 s, the temperature barely reaches 40 °C. These slightly elevated temperature values, compared to those reported by Museux et al., are attributed to the inclusion of the thermal relaxation time parameter in the model. This parameter varies depending on tissue type [47] and was introduced to account for the non-uniform temperature distribution resulting from the non-homogeneous internal structure of biological tissues.

The presence of thermal relaxation time implies a deviation from classical Fourier heat conduction, indicating non-Fourier behavior, which may lead to non-uniform thermal stress distribution and the perception of intense pain. To enhance simulation accuracy, tissue-specific thermal relaxation times were implemented. As expected, decreasing the thermal relaxation time value leads to increased peak temperatures, in accordance with the literature and kinetic theory [48,49].

In conclusion, incorporating the thermal relaxation time of a specific tissue into the simulation model yields results that more accurately reflect experimental observations.

Additionally, the improved bioheat equation used in this study provides a more accurate representation of laser-induced thermal responses compared to the classical Pennes equation. Notably, when the thermal relaxation time is neglected, the improved bioheat equation reduces to the traditional Pennes model, which assumes instantaneous heat propagation—a limitation that can lead to overestimation of thermal diffusion. This underscores the importance of incorporating thermal relaxation effects for realistic and precise modeling of laser-tissue interactions.

## 4. Mathematical Validation and Discussion of the Proposed Model

Due to the scarcity and incompleteness of available experimental data [45,50,51], mathematical validation of the proposed model was conducted by comparing a numerical model developed using COMSOL 5.3 with an analytical model constructed in MATHEMATICA 11.0. The thermal distributions obtained in COMSOL (3D numerical simulations) were generated using the Finite Difference Method within the Heat Transfer Module. In parallel, the corresponding 2D analytical simulations in MATHEMATICA employed the Operator Method to solve the bioheat equation.

It is important to note that simulation serves as a fully justified preliminary step, enabling efficient parameter optimization and feasibility evaluation before engaging in resource-intensive experimental investigations.

All COMSOL simulations were conducted using the non-Fourier bioheat equation model. The laser energy deposition was modeled as a spatial Dirac delta function centered at the laser focus (*x* = 0), while the temporal profile of the laser exposure was defined using finite pulse durations of 60 s and 120 s. It should be noted that at *t* = 0 (prior to laser activation), the temperature distribution is set to physiological normothermia (36–37 °C), representing a stable pre-treatment baseline. During the modeled exposure periods, local heating and perfusion dominate thermal evolution at the treatment site. Consequently, systemic thermoregulatory responses—particularly those mediated by the hypothalamus—are unlikely to have a significant impact on the immediate thermal behavior at the target location.

It is important to mention that the selection of exposure times in laser-based cancer therapies is critical for achieving effective thermal ablation while minimizing damage to surrounding healthy tissues. Thus, in our validation simulations, these two specific timeframes (i.e., 60 s and 120 s) were deliberately chosen to reach therapeutic temperatures exceeding 70 °C, a threshold commonly associated with coagulative necrosis (a primary mechanism for tumor destruction) [44], irreversible protein denaturation, and tumor tissue ablation. Such durations are representative of clinical protocols used in laser-based cancer therapy, where the goal is to selectively destroy malignant skin and subcutaneous tissues while minimizing collateral damage to surrounding healthy structures. In this context, it was indicated that the extended exposure times, which are orders of magnitude longer than the typical thermal relaxation times in biological tissues (generally in the microsecond to millisecond range), were aimed at targeting and damaging larger tissue regions, including those with low melanin content [42,43,52]. Under these conditions—characterized by moderate heating rates and macroscopic thermal diffusion timescales—the contribution of *τ*_q_ to the overall thermal field becomes negligible.

It was assumed that the boundary of the diseased tissue has a radius of 10 mm, while the total radius of the investigated tissue region is 30 mm. The simulation accounts for the wavelength-dependent absorption coefficient and applies a laser power density (also referred to as irradiance) of 10^7^ W·m^−3^. This value was chosen to ensure that peak temperatures exceeding 70 °C within the targeted tissue volume—corresponding to the threshold for coagulative necrosis, the primary mechanism underlying effective laser-based thermal ablation. One should note that the selectivity of tissue removal is directly influenced by the tuning of both laser irradiance and exposure duration. Inadequate power densities (<10^7^ W/m^3^) may result in sub-therapeutic temperatures, leading to incomplete ablation, while excessively high values (>10^8^ W/m^3^) may cause uncontrolled overheating and collateral damage beyond the target site. Accordingly, the value of 10^7^ W/m^3^ was selected as an optimal irradiance level for simulating efficient and selective ablation of soft biological tissue.

A laser wavelength of 800 nm was selected, corresponding to near-infrared diode lasers commonly used in medical procedures such as phototherapy, dental treatments, and soft tissue applications.

The laser pulse duration, material response time, and characteristic thermal relaxation time in our case do not require the introduction of a finite delay in the source term. This modeling approach is consistent with previously published studies addressing similar irradiation conditions [53].

In our COMSOL Multiphysics simulations, we implemented a finite spatial beam profile corresponding to typical clinical parameters (Gaussian-like, ~2 mm spot size) to rectify the influence of the laser beam on the precision of the ablation margins. We also ensured that the computational domain was significantly larger than the beam diameter (>10 mm), to avoid artificial thermal reflections or edge effects that could distort the temperature field.

**Figure 7:**
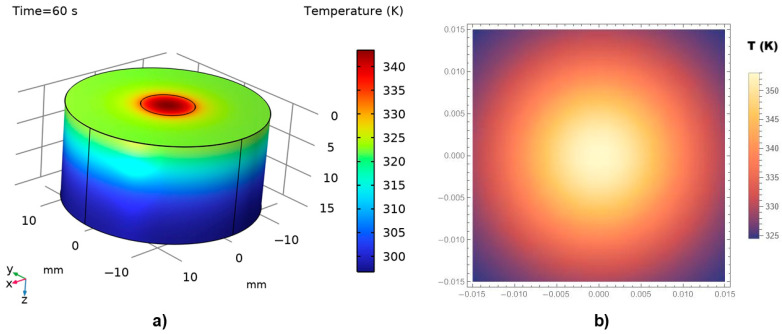
Thermal field distribution in irradiated diseased tissue and adjacent healthy tissue for a thermal exposure time of 60 s: (**a**) 3D numerical boundary representation obtained using COMSOL Multiphysics, (**b**) 2D analytical temperature distribution computed with MATHEMATICA.

Figure 7a,b illustrate the thermal field distribution obtained from the numerical (COMSOL) and analytical (MATHEMATICA) simulations, respectively, for a thermal exposure time of 60 s.

A reasonable agreement is observed between the two modeling approaches. As expected, in both cases, the maximum temperature—above 70 °C (345–350 K)—is recorded at the point of laser incidence on the tissue surface. The temperature then decreases sharply in the surrounding regions, with the majority of the tissue exposed to temperatures below 45 °C. Coagulative necrosis typically occurs when tissue temperatures reach approximately 60 °C due to protein denaturation. However, the extent of thermal damage depends not only on the temperature achieved but also on the duration of exposure [54]. The relationship between temperature and exposure time is often described by the Arrhenius equation, which models the rate of thermal damage accumulation in tissues. For instance, exposure to 50 °C for 4–6 min can induce tissue coagulation, while higher temperatures require shorter exposure times to achieve similar effects [44].

The COMSOL 3D simulation also reveals a gradual decrease in temperature across the tissue layers beneath the directly irradiated surface. Normal physiological temperature is re-established at a depth of several millimeters, indicating the limited thermal penetration under the given irradiation conditions.

It is important to note that, despite differences in dimensionality (i.e., 3D vs. 2D), the temperature contour trends are highly similar, suggesting that the analytical model effectively captures the essential physics represented in the numerical solution.

**Figure 8:**
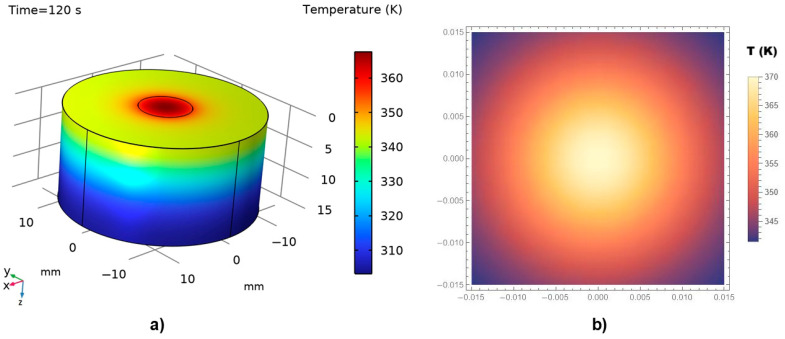
Thermal field distribution in irradiated diseased tissue and adjacent healthy tissue for a thermal exposure time of 120 s: (**a**) 3D numerical boundary representation obtained using COMSOL Multiphysics, (**b**) 2D analytical temperature distribution computed with MATHEMATICA.

The simulations presented in Figure 8, corresponding to a doubled thermal exposure time of 120 s, exhibit a behavior similar to that shown in Figure 7. However, a higher temperature distribution is observed, reaching values close to 100 °C. Both the penetration depth and the thermal field exhibit significant, non-linear variations with respect to the thermal exposure time.

An important observation from Figure 7 and Figure 8 is that the temperature at the irradiation site increases progressively with exposure time and continues to rise until the laser is turned off. Consequently, the extent of thermal damage—reflected in cell destruction or tissue ablation—is directly proportional to the duration of laser exposure. Despite the differences in graphical presentation, Figure 7 and Figure 8 are consistent in depicting the corresponding physical phenomena, particularly in representing the temperature distribution at the laser-tissue interface. In this respect, due to differences in rendering capabilities and scaling conventions between COMSOL and MATHEMATICA, it was challenging—if not impossible—to apply an identical temperature scale across both software platforms. Nevertheless, we would like to stress that the underlying concepts and graphical representations are scientifically sound and accurately reflect the thermal behavior modeled.

In our validation simulations, the systematic analysis of laser exposure durations ranging from 0.01 to 120 s was performed. The obtained results clearly indicate that exposure time plays a dominant role in determining both (i) the depth and lateral extent of thermal propagation, and (ii) the likelihood of thermal injury, including superficial skin burns and cosmetic effects.

At very short exposure durations (0.01–1 s), heat remains highly localized at the laser focus, with thermal penetration typically limited to a few hundred micrometers to approximately one millimeter. In this regime, the risk of collateral damage to surrounding skin is minimal, assuming that the pulse energy and spot size are appropriately controlled.

In contrast, at moderate to long exposure durations (60 s to 120 s), thermal diffusion becomes substantial, allowing heat to propagate both deeper and laterally into the surrounding tissue. Under such conditions, the thermal propagation distances can extend over several millimeters, and peak temperatures exceeding 70–100 °C can occur not only at the laser focal point but also in adjacent tissue layers. Thus, precise control of exposure temperatures—achieved by adjusting laser intensity, exposure duration, and spot size—allows for significantly improved mastery of local thermal effects.

In contrast, inadequate temperature control may result in: (i) under-treatment, due to sub-ablative temperatures, which fail to achieve necrosis, or (ii) overheating (temperatures exceeding 120 °C), which can cause undesirable effects such as carbonization, blister formation, and impaired wound healing due to excessive collateral damage. Therefore, the incorporation of real-time temperature monitoring and precise thermal dosimetry—informed by validated thermal models such as the one developed in this study—is critical for enhancing both the efficacy and safety of laser-based therapies.

In our simulations, we observed that exposure durations ≥ 60 s can result in lateral thermal spread of 4–6 mm or more, with tissue volumes reaching critical thermal thresholds (>50–60 °C for protein denaturation; >70 °C for coagulative necrosis). Notably, extended exposures increase the risk of thermal injury to superficial skin structures, including blistering, scarring, and pigmentation changes, particularly when the treatment zone is near the skin surface. These risks are well documented in clinical applications involving long-pulse laser ablation [55]. These findings emphasize the need for careful treatment planning, incorporating proper laser parameter selection, cooling mechanisms, and targeting strategies to avoid unintended injury to non-targeted tissues.

The efficacy of laser ablation therapy depends on various parameters, including laser power, beam distribution, and tissue properties. Thermomechanical modeling studies have demonstrated that optimizing these parameters is essential for achieving the desired temperature distribution and maximizing the fraction of necrotic tissue within the target volume. By selecting appropriate thermal exposure times, clinicians can tailor the thermal dose to the specific characteristics of the tumor, thereby enhancing treatment outcomes [56].

However, some physical limitations of the model could be identified, and we would like to clearly state the following:

(i) The thermal transport is governed by an extended Pennes bioheat equation implemented in a hyperbolic (telegraph-type) formulation, incorporating a finite thermal relaxation time (*τ_q_* = 1.16 s or 2.61 s), thereby enabling finite-speed thermal wave propagation, and improving physical realism over the classical Fourier model;
(ii) The model assumes constant tissue properties (thermal conductivity, heat capacity, and perfusion rate), and does not incorporate temperature-dependent variations, phase transitions (e.g., vaporization of water), or dynamic damage evolution during heating;
(iii) The temperature predictions are valid up to approximately 100 °C. Beyond this range, nonlinear and irreversible phenomena—such as boiling, carbonization, explosive vaporization, and structural disintegration of tissue—are expected to occur but are not captured in the present formulation;
(iv) The temporal validity of the model extends over exposure durations from 0.01 s to 120 s, as tested in this study. For ultrashort laser pulses (nanoseconds to microseconds), additional physical effects—such as electron-phonon coupling and non-equilibrium heat transfer—would become relevant and lie beyond the scope of this study;
(v) The model is spatially valid at length scales larger than the optical mean free path (typically tens to hundreds of micrometers), consistent with the macroscopic assumptions of the Pennes framework. At sub-micron scales, microstructural heterogeneities and ballistic photon transport effects would need to be considered.

In summary, the model employed in this study provides a physically consistent and computationally efficient framework for simulating laser-induced thermal transport in biological tissues, within the clinically relevant ranges of temperature (≤100 °C), exposure time (0.01–120 s), and spatial scale relevant to clinical laser ablation treatments.

## 5. Conclusions

A three-dimensional analytical model is developed using an extended version of the Pennes bioheat equation formulated in a non-Fourier framework to describe the temperature distribution resulting from laser-tissue interaction. This model aims to extend and refine the classical bioheat equation by incorporating Dirac delta functions (to mathematically represent instantaneous localized point-source heating, such as that generated by focused laser irradiation of a small tissue volume) and the telegraph equation (introducing the finite temporal response of heat flux), thereby enhancing the accuracy of heat transfer predictions in diseased skin tissue and adjacent subcutaneous tissues.

This approach was selected to overcome the limitations of the classical Fourier model —in particular, its prediction of infinite heat propagation speed, which is physically unrealistic for localized and transient thermal processes such as laser-based heating of biological tissue. In this regard, a finite thermal relaxation time (*τ_q_*) is introduced, which accounts for the delay between the imposed temperature gradient and the resulting heat flux. This model describes thermal wave propagation at a finite speed, resulting in a more realistic description of spatiotemporal temperature distributions during short or highly localized heating events.

This study systematically analyzed the relationship between *τ_q_* and the resulting spatiotemporal temperature field. Our simulation results demonstrate that the inclusion and optimization of *τ_q_*—set to 1.16 s for the specific tissue and laser parameters studied—significantly influenced: (i) the spatial precision of the resulting thermal field, (ii) the rate of temperature rise, (iii) the extent of lateral thermal diffusion, and (iv) the model’s ability to prevent undesirable effects, such as overheating or insufficient ablation. By incorporating *τ_q_*, the model yields more accurate and physically realistic temperature predictions, particularly under conditions involving short laser pulses, small beam diameters, or selective lesion targeting. This enhanced accuracy facilitates more effective clinical parameter selection—including laser power, exposure duration, and repetition rate—and supports: (i) optimization of therapeutic efficacy, (ii) improved selectivity in tissue ablation, and (iii) reduced collateral damage to surrounding healthy tissues.

It is also important to mention that a mathematical validation of the simulation results was performed by comparing the COMSOL-based numerical simulations—employing the Finite Difference Method—with the analytical solutions obtained using the Operator method in MATHEMATICA. It is widely recognized that COMSOL is a robust and versatile platform capable of accurately reproducing real experimental conditions, thereby reinforcing the reliability of the simulation outcomes presented in this study. The current study holds not only theoretical significance but also practical relevance, providing simulation results that can inform laser-based medical procedures and diagnostic measurements.

Future refinement of the present model may be provided by prospective research in the following areas: (i) integration of thermal damage kinetics, (ii) incorporating temperature-dependent thermal and optical parameters (i.e., thermal conductivity, specific heat capacity, blood perfusion, scattering, and absorption coefficients), (iii) the inclusion of dynamic blood perfusion effects, (iv) incorporation of experimentally measured laser beam profiles, (v) multi-scale modeling approaches (coupling micro-scale optical transport with macro-scale thermal responses), and (vi) considering the anisotropic nature of skin, particularly in relation to the orientation of collagen fibers and their biomechanical implications.

## Figures and Tables

**Table 1:** Main physical parameters used in all computational analyses.

Properties	Units	Skin	Ref.
Tissue temperature, T	°C	37	
Tissue density, *ρ*	kg m^−3^	1180	[39]
Blood density, *ρ_b_*	kg m^−3^	1060	[24]
Tissue-specific heat, *c*	J (kg^−1^K^−1^)	2291	[1]
Blood perfusion rate, *ω_b_*	Kg (m^−3^s^−1^)	0.5	[40,41]
Blood temperature, *Tb*	°C	38	[41]
Thermal relaxation time, $\tau_{q}$	s	0.01–10.44	[38]
Tissue thermal conductivity, *k*	W (m^−1^K^−1^)	0.58	[39]
Metabolic heat generation rate, *Q_met_*	W m^−3^	33,800	[1]
Target diameter, *d*	mm	2, 3	
Laser power density, *P*	W m^−3^	10^7^	[1]
Thermal exposure time, t	s	0.01–120	[42,43]
Laser wavelength, λ	nm	800	[25,44]

**Table 2:** Key differences between the simulation scenarios presented in Figure 1, Figure 2, Figure 3, Figure 4, Figure 5 and Figure 6.

Figure No.	Target Diameter, d [mm]	Spatial Coordinates [m]	Thermal Exposure Time, t [s]	Thermal $\mathrm{Relaxation}\;\mathrm{Time},\;\tau_{q}$ [s]
1	2	*y* = *z* = 0	30	0
2	2	*z* = 0	0.007	1.16
3	3	*z* = 0	0.007	2.61
4	3	*z* = 0	0.01	2.61
5	3	*z* = 0	0.01	0.01
6	2	*z* = 0.001	0.01	1.16

## Data Availability

The original contributions presented in this study are included in the article. Further inquiries can be directed to the corresponding author.
